# Afatinib for the Treatment of Non-Small Cell Lung Cancer Harboring Uncommon *EGFR* Mutations: An Updated Database of 1023 Cases Brief Report

**DOI:** 10.3389/fonc.2022.834704

**Published:** 2022-04-28

**Authors:** James Chih-Hsin Yang, Martin Schuler, Sanjay Popat, Satoru Miura, Keunchil Park, Antonio Passaro, Filippo De Marinis, Flavio Solca, Angela Märten, Edward S. Kim

**Affiliations:** ^1^Department of Medical Oncology, National Taiwan University Cancer Center and Department of Oncology, National Taiwan University Hospital, Taipei, Taiwan; ^2^Department of Medical Oncology, University Hospital Essen, West German Cancer Center Essen, Essen, Germany; ^3^Lung Unit, Royal Marsden National Health Service Foundation Trust, London, United Kingdom; ^4^The Institute of Cancer Research, London, United Kingdom; ^5^Department of Internal Medicine, Niigata Cancer Center Hospital, Niigata, Japan; ^6^Division of Hematology/Oncology, Samsung Medical Center, Sungkyunkwan University School of Medicine, Seoul, South Korea; ^7^Division of Thoracic Oncology, European Institute of Oncology (IEO) IRCCS, Milan, Italy; ^8^Boehringer Ingelheim RCV GmbH & Co KG, Vienna, Austria; ^9^Boehringer Ingelheim International GmbH, Ingelheim am Rhein, Germany; ^10^City of Hope National Medical Center, Los Angeles, CA, United States

**Keywords:** afatinib, non-small-cell lung cancer, uncommon *EGFR* mutations, compound mutations, *EGFR* exon 20 insertions

## Abstract

**Introduction:**

Previously, we developed a database of 693 patients with NSCLC and uncommon *EGFR* mutations treated with afatinib. Here, we provide an update of >1000 patients, with more data on specific mutations.

**Methods:**

Patients were identified from a prospective database developed by Boehringer Ingelheim and *via* literature review. Mutations were categorized as T790M-positive, exon 20 insertions, major uncommon (G719X, L861Q, S768I) and ‘others’. Patients with compound mutations (≥2 *EGFR* mutations) were analyzed separately. Key endpoints were time to treatment failure (TTF) and objective response rate (ORR).

**Results:**

Of 1023 patients included, 587 patients were EGFR TKI-naïve and 425 were EGFR TKI-pretreated. The distribution of mutation categories was: major uncommon (41.4%); exon 20 insertions (22.3%); T790M (20.3%); and ‘others’ (15.9%); 38.6% had compound mutations. Overall, median TTF (TKI naïve/pretreated) was 10.7 and 4.5 months. ORR was 49.8% and 26.8%, respectively. In TKI-naïve patients, afatinib demonstrated activity against major uncommon mutations (median TTF: 12.6 months; ORR: 59.0%), ‘other’ mutations (median TTF: 10.7 months; ORR: 63.9%) including strong activity against E709X (11.4 months; 84.6%) and L747X (14.7 months; 80.0%), and compound mutations (11.5 months; 63.9%). Although sample sizes were small, notable activity was observed against specific exon 20 insertions at residues A763, M766, N771, and V769, and against osimertinib resistance mutations (G724S, L718X, C797S).

**Conclusion:**

Afatinib should be considered as a first-line treatment option for NSCLC patients with major uncommon, compound, ‘other’ (including E709X and L747X) and some specific exon 20 insertion mutations. Moderate activity was seen against osimertinib resistance *EGFR* mutations.

## Introduction

In head-to-head clinical trials, afatinib, dacomitinib and osimertinib have all demonstrated superiority to first-generation epidermal growth factor receptor (EGFR) tyrosine kinase inhibitors (TKIs) in patients with *EGFR* mutation-positive non-small cell lung cancer (NSCLC) (1–3). However, these studies were exclusively undertaken in patients with tumors harboring common *EGFR* mutations (exon 19 deletions [Del19] and the L858R mutation in exon 21). Therefore, few prospective data are available to help inform treatment decisions for patients with tumors harboring uncommon *EGFR* mutations. In general, preclinical studies (4–6) and retrospective clinical data (7) indicate that second- and third-generation TKIs have broader activity across uncommon mutations than first-generation agents. However, as uncommon *EGFR* mutations are highly heterogeneous it is difficult to know which EGFR TKI is the best option for specific uncommon mutations. There is a largely unmet need for robust clinical data.

We recently constructed a searchable database of 693 NSCLC patients with tumors harboring uncommon *EGFR* mutations treated with afatinib (www.uncommonEGFRmutations.com) (8). Here, we describe an updated analysis of the database that now includes over 1,000 patients, including data on specific uncommon mutations.

## Methods

Methodology has been previously described (8). In brief, patients were identified from a prospective database developed by Boehringer Ingelheim and *via* literature review.

Central mutation testing was only performed in patients enrolled in the LUX-Lung trials (9). In all other patients, mutation detection was undertaken locally using different methodologies. Mutations were categorized into four groups: i) T790M; ii) exon 20 insertions; iii) ‘major’ uncommon mutations; iv) ‘other’ uncommon mutations. Compound mutations, defined as cases where at least two *EGFR* mutations were present (at least one of which was an uncommon mutation), were analyzed separately.

The key endpoints were objective response rate (ORR) and time to treatment failure (TTF), defined as time from start of therapy to treatment discontinuation for any reason, or death. Apart from the LUX-Lung studies, tumor response was assessed by the treating investigator by local assessment. TTF was calculated using Kaplan–Meier estimates. A Cox proportional hazards model was used to calculate hazard ratios and 95% confidence intervals (CIs). There was no formal statistical analysis plan.

## Results

### Patients

A total of 1,023 patients (EGFR TKI-naïve: n = 587; EGFR TKI-pretreated: n = 425) were included (**Supplementary Figure 1**). Patient demographics are shown in **Supplementary Table 1**. The source of 693 patients has been previously reported (8). The source references for the additional 330 patients are listed in the **Supplementary Appendix**.

**Figure 1:**
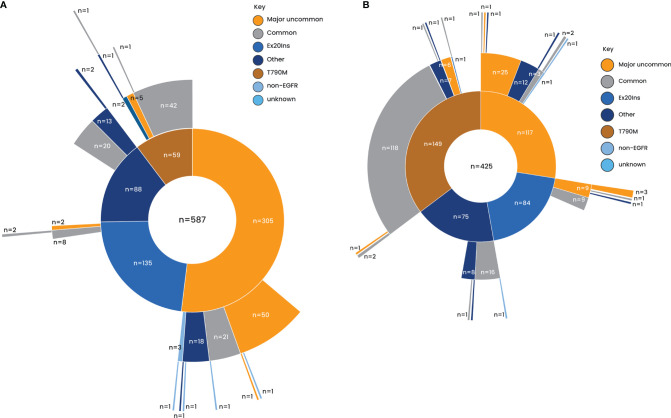
Distribution of uncommon mutations. **(A)** EGFR TKI naïve. **(B)** EGFR TKI pretreated. EGFR, epidermal growth factor receptor; TKI, tyrosine kinase inhibitor.

Overall, 41.4% of patients had a tumor harboring a major uncommon mutation, 22.3% had an exon 20 insertion (of which only 18.4% were fully informative), 20.3% had a T790M mutation (predominantly in the EGFR TKI-pretreated patients), and 15.9% had other uncommon *EGFR* mutations (**Supplementary Table 2**). Overall, 38.6% of patients had a compound mutation. In both TKI-naïve and EGFR TKI-pretreated patients, compound mutations were more common in the T790M category and less common in the exon 20 insertion category (**Figure 1**).

### Updated Time to Treatment Failure and Tumor Response

Overall, median TTF was 10.7 months (95% CI: 9.7–11.5) in EGFR TKI-naïve patients and 4.5 months (95% CI: 3.9–5.6; **Table 1** and **Supplementary Figure 2A**) in EGFR TKI-pretreated patients. Median TTF was similar regardless of ethnicity (**Supplementary Figure 2B**). Median TTF in patients with confirmed brain metastases (56% major uncommon, 25% exon 20 insertions, 9% T790M and 10% others) was 8.2 months (**Supplementary Figure 2C**). In EGFR TKI-naïve patients, median TTF was 12.6 months (95% CI: 11.5–15.9) in patients with major uncommon mutations, 10.7 months (95% CI: 7.0–12.0; **Table 1** and **Supplementary Figure 2D**) in patients with ‘other’ uncommon mutations and 11.5 months (95% CI: 9.5–13.8; **Table 1**) in patients with compound mutations. The large sample size in this study facilitated meaningful analysis of TTF with afatinib against specific uncommon mutations, including the major uncommon mutations, G719X (median 14.2 months), L861Q (median 11.5 months), S768I (median 15.9 months) and the ‘other’ uncommon mutations, E709X (median 11.4 months) and L747X (median 14.7 months; **Table 1**).

**Table 1:** TTF and ORR with afatinib in patients with NSCLC harboring uncommon mutations.

	TTF				ORR			
	n	EGFR TKI Naïve	n	EGFR TKI Pretreated	n	EGFR TKI Naïve	n	EGFR TKI Pretreated
Overall	587	10.7 (9.7–11.5)	425	4.5 (3.9–5.6)	506	252 (49.8)	205	55 (26.8)
Major uncommon mutation	305	12.6 (11.5–15.9)	117	5.3 (3.6–8.4)	278	164 (59.0)	54	16 (29.6)
G719X	194	14.2 (11.5–17.0)	81	4.7 (3.0–8.9)	181	111 (61.3)	39	5 (12.8)
L861Q	109	11.5 (10.5–13.8)	45	4.4 (2.5–8.1)	97	56 (57.7)	23	9 (39.1)
S768I	61	15.9 (11.5–20.5)	34	3.0 (3.0–5.7)	56	40 (71.4)	16	3 (18.8)
Compound	182	11.5 (9.5–13.8)	210	4.4 (3.5–5.6)	155	99 (63.9)	110	24 (21.8)
+ major uncommon	90	16.0 (14.2–20.5)	38	6.0 (3.0–9.9)	83	61 (73.5)	20	5 (25.0)
+ exon20ins	11	12.5 (3.8–13.1)	19	4.2 (2.0–7.5)	9	5 (55.6)	10	0
+ T790M	48	4.7 (3.0–6.5)	131	3.8 (3.0–5.7)	35	11 (31.4)	62	12 (19.4)
+ others	33	11.5 (9.5–13.8)	22	4.5 (2.7–15.0)	28	22 (78.6)	18	7 (38.9)
Exon 20 insertion	135	5.7 (4.8–8.3)	84	4.4 (2.8–7.5)	114	31 (27.2)	31	4 (12.9)
T790M	59	4.7 (2.8–6.5)	149	4.0 (3.4–5.6)	42	11 (26.2)	73	13 (17.8)
Others	88	10.7 (7.0–12.0)	75	4.5 (3.6–8.1)	72	46 (63.9)	47	22 (46.8)
E709X	15	11.4 (3.8–19.3)	11	12.2 (7.0–NE)	13	11 (84.6)	7	6 (85.7)
L747X	18	14.7 (9.0–19.8)	4	NE (0.5–NE)	15	12 (80.0)	3	2 (66.7)

EGFR, epidermal growth factor receptor; NE, not evaluable; NSCLC, non-small-cell lung cancer; ORR, objective response rate; TKI, tyrosine kinase inhibitors; TTF, time to treatment failure.

Overall, 252 EGFR TKI-naïve patients (49.8%) responded to treatment (**Table 1**). The ORR was 53.8% in Asians, 43.4% in non-Asians and 43.9% in patients with confirmed brain metastases. ORR was higher in patients with ‘other’ uncommon mutations (63.9%) and major uncommon mutations (59.0%). Moderate activity was observed in patients with exon 20 insertions (27.2%) and those with T790M (26.2%). High response rates were observed in patients with the specific uncommon mutations, G719X (61.3%), L861Q (57.7%), S768I (71.4%), E709X (84.6%) and L747X (80.0%). In patients with compound mutations, ORR was 63.9%.

### Outcomes in Patients With Specific Fully-Defined Exon 20 Insertions

In the 42 patients with informative exon 20 insertions, median TTF was 9.1 months (95% CI: 7.4–14.2). Median TTF was 9.1 months in EGFR TKI-naïve patients and 10.8 months in EGFR TKI-pretreated patients. ORR was 48% and 17%, respectively (**Table 2**). In terms of individual mutation types, insertions at amino acid A763, M766, N771 and V769 showed evidence of sensitivity to afatinib, with TTF ranging from 8.0 to 39.0 months and ORRs ranging from 50 to 100% (**Table 2**).

**Table 2:** TTF, ORR and DCR in patients with NSCLC harboring fully-defined exon 20 insertion mutations.

Exon 20 insertion type	n (%)	Median TTF, months (95% CI)	ORR, %	DCR, %
All informative exon 20 insertions	42 (100)	9.1 (7.4–14.2)	33	76
EGFR TKI naïve	23 (54.8)	9.1 (5.2–14.2)	47	79
EGFR TKI pretreated*^a^*	11 (26.2)	10.8 (5.3–36.0)	17	67
A763_Y764insFQEA; A763_V765dup	4 (9.5)	39.0 (8.2–39.0)	50	100
A767_S768insSVA; _V769dup/ASV; insASVD	5 (11.9)	3.7 (1.0–36.0)	0	75
D770_N771insGL/SVD	3 (7.1)	3.8 (3.0–20.1)	0	33
H773_R776insYNPY; _V774dup/insH; dup	9 (21.4)	24.0 (6.1–NE)	0	71
M766delinsMATL; insASV	2 (4.8)	12.9 (11.6–14.2)	100	100
N771_H773dup; _772insPHGH; delinsKG; _P772insGY	7 (16.7)	10.0 (5.2–NE)	71	100
S768_D770dup	5 (11.9)	8.5 (NE–NE)	0	25
V769_770INSV; _D770insASV/GVV	7 (16.7)	8.0 (1.2–14.3)	75	100

CI, confidence interval; DCR, disease control rate; EGFR, epidermal growth factor receptor; NE, not evaluable; NSCLC, non-small-cell lung cancer; ORR, objective response rate; TKI, tyrosine kinase inhibitors; TTF, time to treatment failure. ^a^A767: n = 2; H773: n = 3; N771: n = 1; S768: n = 2; V769: n = 3.

### Outcomes in Patients With Uncommon *EGFR* Mutations Associated With Acquired Resistance to Osimertinib

A total of 19 patients who were treated with afatinib had uncommon *EGFR* mutations that are associated with acquired resistance to osimertinib (G724S [n = 13], L718Q [n = 3], L718V [n = 2], C797S [n = 1]; **Table 3**). Fifteen of these patients received osimertinib up front.

**Table 3:** Outcomes in patients with NSCLC harboring uncommon *EGFR* mutations which are known to be resistance mechanisms to osimertinib.

Ethnicity	Gender	Age	Smoking status	Brain metastases	*EGFR* mutations			Afatinib treatment setting	Best response	TTF, months
Asian	F	55	–	Yes	G724S	Del19	–	Post osimertinib	PR	3.8+
Non-Asian	F	49	NS	Yes	G724S	Del19	–	Post osimertinib	–	3.0 *^a^*
Asian	F	64	NS	–	G724S	R776H	–	TKI naïve	SD	17.0 *^b^*
Asian	–	–	–	–	G724S	E746_S752delinsV	–	Post osimertinib	SD	Median 4.5
Asian	–	–	–	–	G724S	E746_S752delinsV	–	Post osimertinib	SD	
Asian	–	–	–	–	G724S	E746_S752delinsV	–	Post osimertinib	SD	
Asian	–	–	–	–	G724S	S768I	–	Osimertinib naïve	SD	
Asian	–	–	–	–	G724S	S768I	Del19	Osimertinib naïve	SD	
Asian	–	–	–	–	G724S	Exon20ins	–	Osimertinib naïve	SD	
Asian	M	–	–	–	G724S	E746_S752delinsV	–	Post osimertinib	SD	2.7
Asian	F	–	–	–	G724S	E746_S752delinsV	–	Post osimertinib	SD	6.1
Non-Asian	M	49	–	Yes	G724S	Del19	–	Post osimertinib	PR	8.0+ *^a^*
Non-Asian	M	51	NS	Yes	G724S	Del19	–	Post osimertinib	SD	10.0+
Asian	F	65	–	Yes	L718Q	L858R	(BRAF)	Post osimertinib	SD	4.0
–	F	62	S	–	L718Q	L718V	L858R	Post osimertinib	PR	4.5+
Asian	F	69	NS	–	L718Q	L858R	–	Post osimertinib	PR	4.0
Asian	F	65	NS	–	L718V	L858R	–	Post osimertinib	PR	6.0+
–	–	–	–	–	L718V	L858R	–	Post osimertinib	SD	15.0
–	–	–	–	–	C797S	L858R	V765L	Post osimertinib	SD	4.0

EGFR, epidermal growth factor receptor; NS, never smoker; NSCLC, non-small-cell lung cancer; PR, partial response; S, smoker; SD, stable disease; TTF, time to treatment failure.

a Received afatinib combined with osimertinib.

b Received afatinib combined with bevacizumab.

In patients with G724S, ORR was 17% and the disease control rate (DCR) was 100%. TTF ranged from 2.7 months to 17.0 months. In patients with L718Q/V, ORR was 60% and DCR was 100%. TTF ranged from 4.0 to 15.0 months. The patient with a C797S mutations achieved stable disease (SD). Of the 15 patients treated with afatinib post osimertinib, ORR was 36% and DCR was 100%. TTF ranged from 2.7 to 15.0 months. Three patients with G719X received afatinib post osimertinib with best response of partial response (PR), SD and not reported, respectively. TTF ranged from 9.0 to 12.3 months.

## Discussion

This updated analysis demonstrated that NSCLC tumors with certain uncommon mutations respond well to first-line afatinib, with an ORR approaching 50% and median TTF of nearly a year in the overall dataset. In line with previous reports (7–9), activity was higher in patients with major uncommon mutations (with similar response rates against G719X, L861Q and S768I) and ‘other’ uncommon mutations. Activity was observed against specific exon 20 insertion mutations. Compound mutations were relatively common in the database (nearly 40% of cases) and responded well to afatinib; approximately two-thirds of patients responded. Overall, the data demonstrate that afatinib should be considered for the treatment of NSCLC harboring uncommon mutations, depending on the precise nature of the mutation.

The large sample size in this study facilitated the analysis of specific uncommon mutations for which clinical evidence was previously lacking. For example, 26 patients were identified with the exon 18 mutation, E709X, and 22 were identified with the exon 19 mutation, L747X. These mutations responded well to treatment with ORRs of >80%. Interestingly, both E709X and L747X have been identified as pocket volume reducing (PVR) mutations that are predicted to be more sensitive to second-generation EGFR TKIs than first- or third-generation agents (10). We also assessed the activity of afatinib against mutations that have been implicated in the acquired resistance to osimertinib, including G724S (n = 13), L718X (n = 5) and C797S (n = 1), all of which have been identified as PVR mutations (10). G724S mutations showed some sensitivity towards afatinib with an ORR of 17% and DCR of 100%. Time on treatment ranged from 3 to 10 months in patients pretreated with osimertinib. Interestingly, one TKI-naïve patient remained on treatment with afatinib for 17 months. Patients with L718X demonstrated an ORR of 60%. Given the current paucity of targeted treatment options following failure of osimertinib, further clinical assessment of afatinib in this setting is warranted in patients with tertiary *EGFR* mutations.

Although exon 20 insertions are widely considered to be insensitive to EGFR TKIs they are highly heterogeneous; both the position of the insertion and the specific residues that are inserted can have distinct effects on the tertiary structure of EGFR, thus influencing the sensitivity of TKIs. For example, insertions between residues 764 and 770 are thought to have a minimal impact on the EGFR TKI binding domain of the receptor (11). Also, the insertion of FQEA at A763 is thought to elicit structural changes to EGFR that are similar to Del19 mutations and thus remains sensitive to EGFR TKIs (12). Therefore, it is important that the exon 20 insertion mutation category is not considered as a single clinical entity. In this analysis, we identified several specific mutations, particularly at the A763, M766, N771 and V769 residues, that appeared to be sensitive to afatinib. Notably, treatment options have recently become available for patients with exon 20 insertions. The TKI, mobocertinib, and the EGFR MET bispecific antibody, amivantamab, have both been approved by the FDA post-platinum doublet chemotherapy (13, 14). These options have conferred response rates of 28% and 40% respectively (15, 16). It remains to be determined whether afatinib may have a role in this setting in certain patients depending on the precise nature of the mutation.

This analysis has several weaknesses. Seventy-three patients were included from published case studies and 209 from case series where selection criteria were not always fully reported. As positive cases are more likely to be published, response rates with afatinib against uncommon mutation categories may be overestimated. Secondly, central *EGFR* mutation testing was only undertaken in a minority of patients. A wide range of different testing methods would have been used at the local level which may have introduced some unrecognized biases. Also, in many of the EGFR TKI-pretreated cases, it is not documented whether mutations were detected prior to the initial EGFR TKI or afatinib. The database only includes patients treated with afatinib and not other EGFR TKIs. Recent data suggest that osimertinib may also have activity against certain uncommon *EGFR* mutations, although it appears to have limited activity against exon 20 insertions (17, 18).

In conclusion, afatinib should be considered as first-line treatment for patients with major uncommon mutations, compound mutations (other than those containing T790M) and certain ‘other’ uncommon mutations, possibly including PVR mutations. Expanded analysis demonstrated strong activity with afatinib against E709X, L747X and certain exon 20 insertions. Moderate activity was observed against *EGFR* mutations implicated in acquired resistance to osimertinib. The heterogeneity of uncommon mutations and their differential sensitivity to afatinib, and other EGFR TKIs, necessitates an improvement in the detection and reporting of *EGFR* mutations in real-world clinical practice, with specific and precise details of mutations required (e.g., regarding exon 20 insertions).

## Data Availability Statement

The datasets generated and analyzed during the study are available from author AM on reasonable request.

## Author Contributions

Study concept and design: JC-HY, EK. Acquisition, analysis, or interpretation of data: All authors. Drafting and critical revision of the manuscript for important intellectual content: All authors. Study supervision: JC-HY, EK. All authors contributed to the article and approved the submitted version.

## Funding

This study was funded by Boehringer Ingelheim.

## Conflict of Interest

JC-HY reports personal and/or institutional fees from Amgen; personal and institutional fees from AstraZeneca, Boehringer Ingelheim, Novartis, Roche/Genentech, Takeda Oncology, Yuhan Pharmaceuticals; institutional fees from Bayer, Daiichi Sankyo, Eli Lilly, Merck KGaA (Darmstadt, Germany), Merck Sharp & Dohme, JNJ; personal fees from Bristol Myers Squibb, Ono Pharmaceuticals, Pfizer; grants from AstraZeneca. MS reports consultancy fees from Amgen, AstraZeneca, Boehringer Ingelheim, Bristol Myers Squibb, GlaxoSmithKline, Janssen, Merck Serono, Novartis, Roche, Sanofi, Takeda; honoraria for CME presentations from Amgen, Boehringer Ingelheim, Bristol Myers Squibb, Janssen, Novartis; research funding from AstraZeneca, Bristol Myers Squibb. SP has received grant support, honoraria, consulting fees, and travel support from Boehringer Ingelheim; consulting fees and travel support from Bristol Myers Squibb; honoraria and consulting fees from Roche, Takeda, AstraZeneca; honoraria from Chugai Pharma; consulting fees from Novartis, Guardant Health, AbbVie, Pfizer; and consulting fees and travel support from Merck Sharp & Dohme. SM has received honoraria from Chugai Pharma, AstraZeneca, Eli Lilly, Merck Sharp & Dohme, Boehringer Ingelheim, Bristol Myers Squibb, Taiho Pharma, Pfizer. KP has received personal fees from Amgen, Astellas, AstraZeneca, Boehringer Ingelheim, Clovis, Daiichi Sankyo, Eli Lilly, Hanmi, Kyowa Hakko Kirin, Incyte, LOXO, Merck KGaA, Merck Sharp & Dohme, Ono Pharmaceuticals, Novartis, and Roche; and research funding from AstraZeneca and Merck Sharp & Dohme. AP received honoraria for consulting, advisory role or lectures from AstraZeneca, Agilent/Dako, Boehringer Ingelheim, Bristol Myers Squibb, Eli Lilly, Jansenn, Merck Sharp & Dohme, Pfizer and Roche Genentech. FDM has received honoraria or consulting fees from AstraZeneca, Bristol Myers Squibb, Merck Sharp & Dohme and Roche. FS and AM are employees of Boehringer Ingelheim. EK has received personal fees from Boehringer Ingelheim, AstraZeneca, and Genentech.

The authors declare that this study received funding from Boehringer Ingelheim. The sponsors played a role in the collection, management, analysis, and interpretation of the data; preparation, review, or approval of the manuscript; and decision to submit the manuscript for publication and as such are included in the author list.

## Publisher’s Note

All claims expressed in this article are solely those of the authors and do not necessarily represent those of their affiliated organizations, or those of the publisher, the editors and the reviewers. Any product that may be evaluated in this article, or claim that may be made by its manufacturer, is not guaranteed or endorsed by the publisher.
