# Supplementary material for: Afatinib for the Treatment of Non-Small Cell Lung Cancer Harboring Uncommon EGFR Mutations: An Updated Database of 1023 Cases Brief Report
Source: Front Oncol. 2022 Apr 28;12:834704. doi: 10.3389/fonc.2022.834704 (PMC9104339; doi:10.3389/fonc.2022.834704)
Supplement: Supplementary file 1 [file DataSheet_1.docx]

**Supplementary Appendix**

| **SUPPLEMENTARY TABLE 1 \|** Patient demographics. | | | |
| --- | --- | --- | --- |
|  | **All**  **n = 1023** | **EGFR TKI naïve**  **n = 587** | **EGFR TKI pretreated**  **n = 425** |
| Age |  |  |  |
| <75 years | 693 (67.7) | 399 (68.0) | 294 (69.2) |
| ≥75 years | 138 (13.5) | 102 (17.4) | 36 (8.5) |
| Missing | 192 (18.8) | 86 (14.7) | 95 (22.4) |
| Gender |  |  |  |
| Male | 363 (35.5) | 235 (40.0) | 128 (30.1) |
| Female | 500 (48.9) | 268 (45.7) | 232 (54.6) |
| Missing | 160 (15.6) | 84 (14.3) | 65 (15.3) |
| Ethnicity |  |  |  |
| Asian | 394 (38.5) | 345 (58.8) | 43 (10.1) |
| Non-Asian | 254 (24.8) | 204 (34.8) | 50 (11.8) |
| Missing | 375 (36.7) | 38 (6.5) | 332 (78.1) |
| Smoking status |  |  |  |
| Ever smoker | 234 (22.9) | 208 (35.4) | 26 (6.1) |
| Never smoker | 290 (28.3) | 240 (40.9) | 50 (11.8) |
| Missing | 499 (48.8) | 139 (23.7) | 349 (82.1) |
| Confirmed brain metastases | 94 (9.2) | 70 (11.9) | 24 (5.6) |

*EGFR, epidermal growth factor receptor; TKI, tyrosine kinase inhibitors.*

| **SUPPLEMENTARY TABLE 2 \|** Mutation frequencies. | | | |  |
| --- | --- | --- | --- | --- |
| **Mutation category, N (%)** | **All**  **n = 1023** | **EGFR-TKI naïve**  **n = 587** | **EGFR-TKI pretreated**  **n = 425** | |
| Major uncommon mutation | 424 (41.4) | 305 (52.0) | 117 (27.5) | |
| G719X | 275 (26.9) | 194 (33.0) | 81 (19.1) | |
| G719X alone | 202 (19.7) | 145 (24.7) | 57 (13.4) | |
| L861Q | 154 (15.1) | 109 (18.6) | 45 (10.6) | |
| L861Q alone | 122 (11.9) | 90 (15.3) | 32 (7.5) | |
| S768I | 97 (9.5) | 61 (10.4) | 34 (8.0) | |
| S768I alone | 47 (4.6) | 26 (4.4) | 19 (4.5) | |
| Compound | 395 (38.6) | 182 (31.0) | 210 (49.4) | |
| With major uncommon mutation | 130 (12.7) | 90 (15.3) | 38 (8.9) | |
| With Exon 20 insertion | 31 (3.0) | 11 (1.9) | 19 (4.5) | |
| With T790M | 179 (17.5) | 48 (8.2) | 131 (30.8) | |
| With others | 55 (5.4) | 33 (5.6) | 22 (5.2) | |
| Exon 20 insertion | 228 (22.3) | 135 (23.0) | 84 (19.8) | |
| Informative | 42 (4.1) | 23 (3.9) | 11 (2.6) | |
| T790M | 208 (20.3) | 59 (10.1) | 149 (35.1) | |
| Others | 163 (15.9) | 88 (15.0) | 75 (17.6) | |
| E709X | 26 (2.5) | 15 (2.6) | 11 (2.6) | |
| L747X | 22 (2.2) | 18 (3.1) | 4 (0.9) | |

*EGFR, epidermal growth factor receptor; TKI, tyrosine kinase inhibitors.*

**SUPPLEMENTARY FIGURE 1 |** Overview of selection process. BoR, best of response; CUP, compassionate use program; EAP, expanded access program; EGFR, epidermal growth factor receptor; NIS, non-interventional study; TKI, tyrosine kinase inhibitors; TTF, time to treatment failure.

**
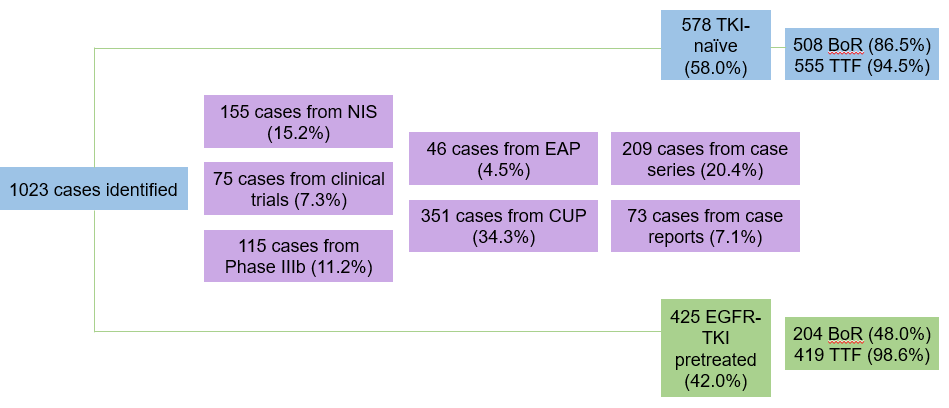
**

**SUPPLEMENTARY FIGURE 2 |** TTF in patients with NSCLC harboring uncommon *EGFR* mutations**.** (*A*) All patients. (*B*) According to ethnicity (EGFR TKI-naïve). (*C*) According to the presence of brain metastases (EGFR TKI-naïve). (*D*) According to mutation category (EGFR TKI-naïve). CI, confidence interval; EGFR, epidermal growth factor receptor; TKI, tyrosine kinase inhibitors; TTF, time to treatment failure.

**A**


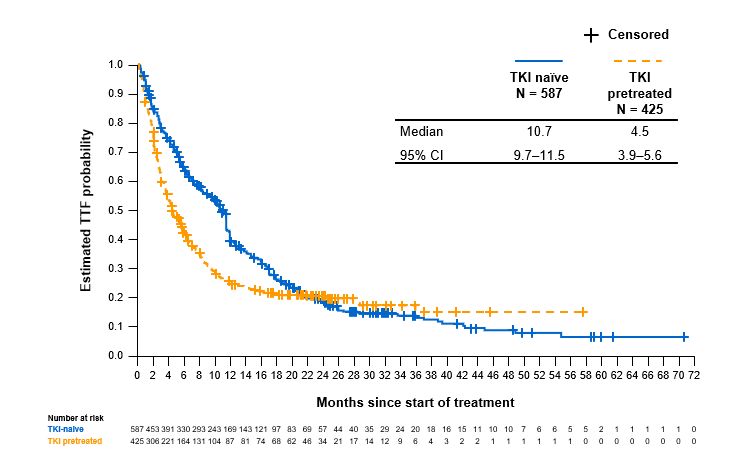


**B**
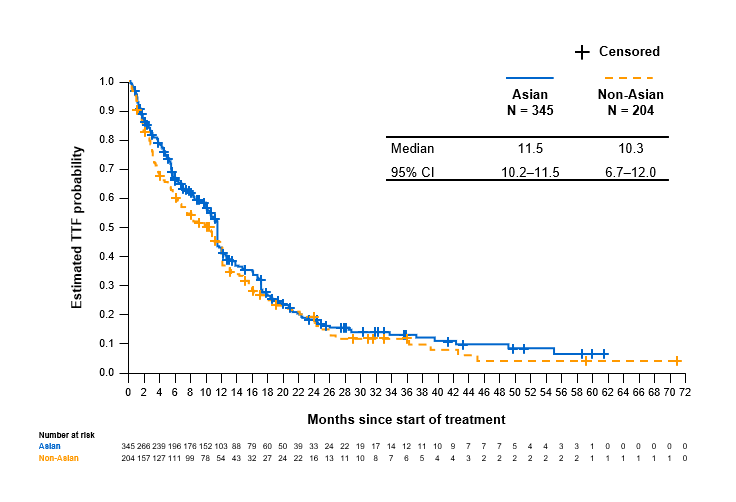


**C**


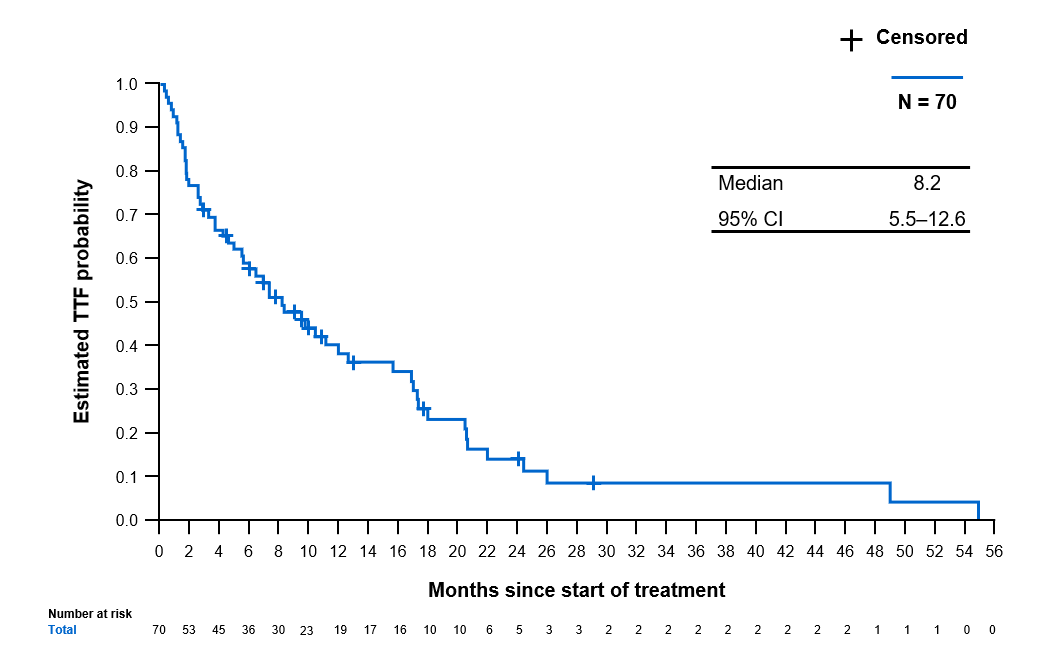


**D**


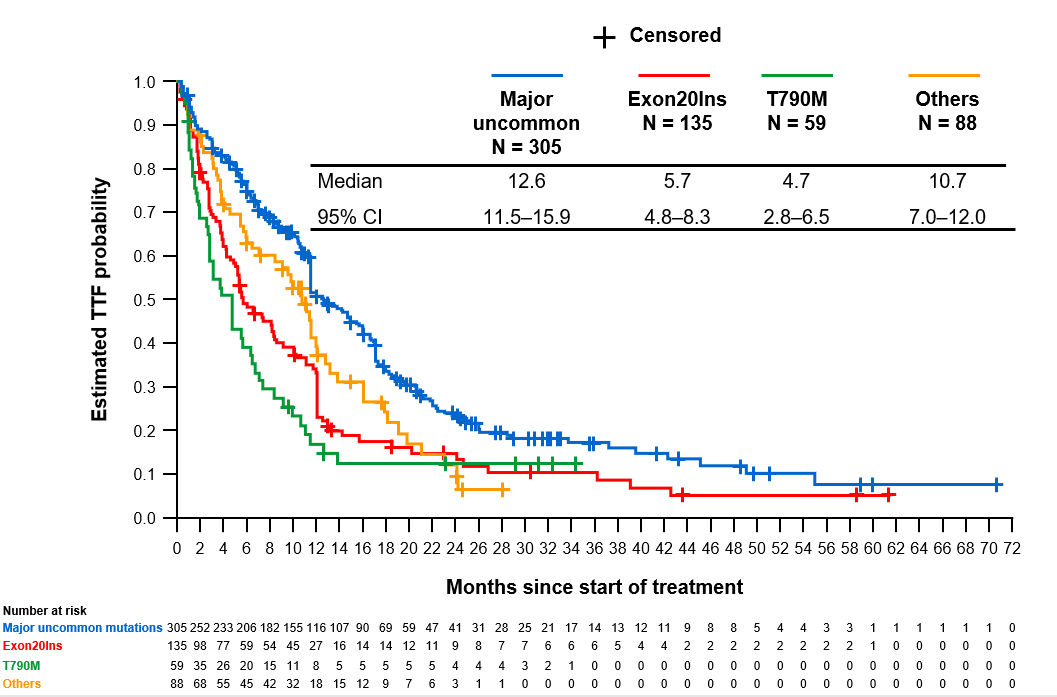


**(47, 52, 6, 56, 1-3, 5, 7, 11, 13-19, 23-27, 30-32, 34, 36, 40, 46, 48-51, 57, 4, 8-10, 12, 20-22, 28, 29, 33, 35, 37-39, 41-45, 53-55)**

**Source References**

Caliman E, Petreni P, Brugia M, Antonuzzo L, Mazzoni F. In regard to "Activity of EGFR TKIs in Caucasian patients with NSCLC harbouring potentially sensitive uncommon EGFR mutations". *Clin Lung Cancer* (2020) 21:e363-e5. doi: 10.1016/j.cllc.2020.02.002.

Chen D, Li XL, Wu B, Zheng XB, Wang WX, Chen HF, et al. A novel oncogenic driver in a lung adenocarcinoma patient harboring an EGFR-KDD and response to afatinib. *Front Oncol* (2020) 10:867. doi: 10.3389/fonc.2020.00867.

Chiang CL, Yeh YC, Chou TY, Chiu CH. Squamous cell carcinoma transformation after acquired resistance to osimertinib in a patient with lung adenocarcinoma harboring uncommon EGFR mutation. *J Formos Med Assoc* (2020) 119:1439-41. doi: 10.1016/j.jfma.2019.12.017.

Citarella F, Russano M, Perrone G, Vincenzi B, Tonini G, Santini D. Response to: successful afatinib rechallenge in a patient with non-small cell lung cancer harboring EGFR G719C and S768I mutations. *Thorac Cancer* (2021) 12:1791-2. doi: 10.1111/1759-7714.13997.

Endo S, Mitsumura T, Ishizuka M, Honda T, R. S, Ikeda S, et al. A case report of a non-small-cell lung cancer patient who was EGFR-negative on a conventional test but was discovered to have an EGFR uncommon mutation on comprehensive genomic profiling and responded to afatinib. *Japan J Lung Canc* (2020) 60:429-33.

Fang W, Gan J, Huang Y, Zhou H, Zhang L. Acquired EGFR L718V mutation and loss of T790M-mediated resistance to osimertinib in a patient with NSCLC who responded to afatinib. *J Thorac Oncol* (2019) 14:e274-e5. doi: 10.1016/j.jtho.2019.07.018.

Fang W, Huang Y, Gan J, Zheng Q, Zhang L. Emergence of EGFR G724S after progression on osimertinib responded to afatinib monotherapy. *J Thorac Oncol* (2020) 15:e36-e7. doi: 10.1016/j.jtho.2019.09.198.

Guo G, Li G, Liu Y, Li H, Guo Q, Liu J, et al. Next-generation sequencing reveals high uncommon EGFR mutations and tumour mutation burden in a subgroup of lung cancer patients. *Front Oncol* (2021) 11:621422. doi: 10.3389/fonc.2021.621422.

He SY, Lin QF, Chen J, Yu GP, Zhang JL, Shen D. Efficacy of afatinib in a patient with rare EGFR (G724S/R776H) mutations and amplification in lung adenocarcinoma: A case report. *World J Clin Cases* (2021) 9:1329-35. doi: 10.12998/wjcc.v9.i6.1329.

Hirose T, Ikegami M, Endo M, Matsumoto Y, Nakashima Y, Mano H, et al. Extensive functional evaluation of exon 20 insertion mutations of EGFR. *Lung Cancer* (2021) 152:135-42. doi: 10.1016/j.lungcan.2020.12.023.

Jiang H, Luo N, Zhang X, Song C, Zang J. EGFR L861Q and CDK4 amplification responding to afatinib combined with palbociclib treatment in a patient with advanced lung squamous cell carcinoma. *Lung Cancer* (2020) 145:216-8. doi: 10.1016/j.lungcan.2020.04.001.

Jiang Y, Zhang J, Jiang X, Cheng L, Liao X, Li Y, et al. Sequential use of EGFR-tyrosine kinase inhibitors based upon EGFR mutation evolution achieves long-term control in a non-small cell lung cancer patient: a case report. *Ann Palliat Med* (2021) 10:7051-6. doi: 10.21037/apm-20-1477.

Kong C, Zhou D, Wu N, Bai C. Multiple intraventricular metastases from lung adenocarcinoma with EGFR G719X mutation: a case report. *BMC Pulm Med* (2020) 20:135. doi: 10.1186/s12890-020-1168-0.

Kunimasa K, Hirotsu Y, Miyashita Y, Goto T, Amemiya K, Mochizuki H, et al. Multiregional sequence revealed SMARCA4 R1192C mutant clones acquired EGFR C797S mutation in the metastatic site of an EGFR-mutated NSCLC patient. *Lung Cancer* (2020) 148:28-32. doi: 10.1016/j.lungcan.2020.07.035.

Kutsuzawa N, Takahashi F, Tomomatsu K, Obayashi S, Takeuchi T, Takihara T, et al. Successful treatment of a patient with lung adenocarcinoma harboring compound EGFR gene mutations, G719X and S768I, with afatinib. *Tokai J Exp Clin Med* (2020) 45:113-6.

Li Y, Lin Y, Wu J, Ye F. Meningeal metastasis patients with EGFR G724S who develop resistance to osimertinib benefit from the addition of afatinib. *Transl Lung Cancer Res* (2020) 9:2188-90. doi: 10.21037/tlcr-20-847.

Lin L, Wu X, Yan S, Zhu Y, Yan Z, Lv D, et al. Response to afatinib in a patient with NSCLC harboring novel EGFR exon 20 insertion mutations. *Onco Targets Ther* (2020) 13:9753-7. doi: 10.2147/ott.S268694.

Long X, Qin T, Lin J. Great efficacy of afatinib in a patient with lung adenocarcinoma harboring EGFR L833V/H835L mutations: a case report. *Onco Targets Ther* (2020) 13:10689-92. doi: 10.2147/ott.S260157.

Long Y, Zhang K, Li Y, Yu M, Zhu J, Huang M. Durable complete response after afatinib and crizotinib in an advanced non-small cell lung cancer patient with EGFR L861Q mutation and acquired MET amplification: a case report. *Ann Palliat Med* (2020) 9:3609-13. doi: 10.21037/apm-19-482.

Longo V, Catino A, Montrone M, Pizzutilo P, Pesola F, Marech I, et al. Successful treatment of triple EGFR mutation T785A/L861Q/H297_E298 with afatinib. *Thorac Cancer* (2021) 12:2031-4. doi: 10.1111/1759-7714.13953.

Lu Z, Wang X, Luo Y, Wei J, Zeng Z, Xiong Q, et al. EGFR (p. G719A+L747V)/EML4-ALK co-alterations in lung adenocarcinoma with leptomeningeal metastasis responding to afatinib treatment: a case report. *Onco Targets Ther* (2021) 14:2823-8. doi: 10.2147/ott.S294635.

Lv D, Lin L, Wu X, Yan S, Ge H, Yan Z, et al. P76.99 Response to afatinib in a patient with NSCLC Harboring novel EGFR exon 20 insertion mutations. *J Thorac Oncol* (2021) 16:S634. doi: 10.1016/j.jtho.2021.01.1156.

Ma C, Liu M, Mu N, Li J, Li L, Jiang R. Efficacy of afatinib for pulmonary adenocarcinoma with leptomeningeal metastases harboring an epidermal growth factor receptor complex mutation (exon 19del+K754E): A case report. *Medicine (Baltimore)* (2020) 99:e22851. doi: 10.1097/md.0000000000022851.

Ma C, Wang S, Mu N, Li J, Liu M, Li L, et al. Effective treatment with afatinib of lung adenocarcinoma with leptomeningeal metastasis harboring the exon 18 p.G719A mutation in the EGFR gene was detected in cerebrospinal fluid: a case report. *Front Oncol* (2020) 10:1635. doi: 10.3389/fonc.2020.01635.

Ma C, Zhang J, Tang D, Ye X, Li J, Mu N, et al. Tyrosine kinase inhibitors could be effective against non-small cell lung cancer brain metastases harboring uncommon EGFR mutations. *Cancer Mol Target Ther* (2020) 10:1-7. doi: 10.3389/fonc.2020.00224.

Masuda T, Sunaga N, Kasahara N, Takehara K, Yatomi M, Hara K, et al. Successful afatinib rechallenge in a patient with non-small cell lung cancer harboring EGFR G719C and S768I mutations. *Thorac Cancer* (2020) 11:2351-6. doi: 10.1111/1759-7714.13532.

Mehta A, Batra U, Sharma M, Sharma S, Nathany S. Detection of rare targetable EGFR variant in metastatic non-small cell lung carcinoma by next generation sequencing: a case report. *J Clin Diag Res* (2020) 16:6.

Mehta A, Vasudevan S. Rare epidermal growth factor receptor gene alterations in non-small cell lung cancer patients, tyrosine kinase inhibitor response and outcome analysis. *Cancer Treat Res Commun* (2021) 28:100398. doi: 10.1016/j.ctarc.2021.100398.

Minari R, Leonetti A, Gnetti L, Zielli T, Ventura L, Bottarelli L, et al. Afatinib therapy in case of EGFR G724S emergence as resistance mechanism to osimertinib. *Anticancer Drugs* (2021) 32:758-62. doi: 10.1097/cad.0000000000001064.

Moran T, Taus A, Arriola E, Aguado C, Dómine M, Rueda AG, et al. Clinical activity of afatinib in patients with non-small-cell lung cancer harboring uncommon EGFR mutations: a spanish retrospective multicenter study. *Clin Lung Cancer* (2020) 21:428-36.e2. doi: 10.1016/j.cllc.2020.04.011.

Ning M, Chun-hua M, Jin-duo L, Mei L, Lin L, Rong J. Afatinib successfully treated leptomeningeal metastasis of lung adenocarcinoma in a patient with EGFR G719A mutation in detection of cerebrospinal fluid. *Chinese J Contemp Neurol Neurosurg* (2020) 20:551-5.

Ohara G, Okauchi S, Sasatani Y, Shiozawa T, Yamada H, Miyazaki K, et al. Long-term survival with afatinib in a patient with lung adenocarcinoma harboring double uncommon EGFR L861Q and G719X mutations. *In Vivo* (2020) 34:1459-62. doi: 10.21873/invivo.11929.

Oyamada Y, Wada S, Fujimoto K. A case of advanced lung adenocarcinoma harboring an epidermal growth factor receptor (EGFR) exon 20 insertion, D770_N771insSVD. *Gan To Kagaku Ryoho* (2021) 48:845-7.

Patil T, Mushtaq R, Marsh S, Azelby C, Pujara M, Davies KD, et al. Clinicopathologic characteristics, treatment outcomes, and acquired resistance patterns of atypical EGFR mutations and HER2 alterations in stage IV non-small-cell lung cancer. *Clin Lung Cancer* (2020) 21:e191-e204. doi: 10.1016/j.cllc.2019.11.008.

Qian Y, Yu G, Dong L, Zhang J, Wang G. P76.21 EGFR-KDD with Duplication of Exons 18-26 Responding to Afatinib Treatment in a Patient with Lung Adenocarcinoma. *J Thorac Oncol* (2021) 16:S595. doi: 10.1016/j.jtho.2021.01.1078.

Qin Y, Jian H, Tong X, Wu X, Wang F, Shao YW, et al. Variability of EGFR exon 20 insertions in 24 468 Chinese lung cancer patients and their divergent responses to EGFR inhibitors. *Mol Oncol* (2020) 14:1695-704. doi: 10.1002/1878-0261.12710.

Raez LE, Carracedo C, Drusbosky LM, Velez M, Carlisle J, Stinchcombe T. EGFR L718V (+)/T790M (-) as a mechanism of resistance in patients with metastatic non-small-cell lung cancer with EGFR L858R mutations. *Clin Lung Cancer* (2021). doi: 10.1016/j.cllc.2021.03.018.

Sehgal K, Rangachari D, VanderLaan PA, Kobayashi SS, Costa DB. Clinical benefit of tyrosine kinase inhibitors in advanced lung cancer with EGFR-G719A and other uncommon EGFR mutations. *Oncologist* (2021) 26:281-7. doi: 10.1002/onco.13537.

Shijubou N, Sumi T, Kamada K, Sawai T, Yamada Y, Nakata H, et al. Long-term response to afatinib in an elderly patient with uncommon epidermal growth factor receptor mutation-positive lung adenocarcinoma. *Thorac Cancer* (2021) 12:989-92. doi: 10.1111/1759-7714.13869.

Starrett JH, Guernet AA, Cuomo ME, Poels KE, van Alderwerelt van Rosenburgh IK, Nagelberg A, et al. Drug sensitivity and allele specificity of first-line osimertinib resistance EGFR mutations. *Cancer Res* (2020) 80:2017-30. doi: 10.1158/0008-5472.Can-19-3819.

Tamiya M, Kunimasa K, Nishino K, Matsumoto S, Kawachi H, Kuno K, et al. Successful treatment of an osimertinib-resistant lung adenocarcinoma with an exon 18 EGFR mutation (G719S) with afatinib plus bevacizumab. *Invest New Drugs* (2021) 39:232-6. doi: 10.1007/s10637-020-00966-7.

Tamura T, Kawakado K, Makimoto G, Nakanishi M, Kuyama S. Limited effect of afatinib in a non-small cell lung cancer patient harboring an epidermal growth factor receptor K860I missense mutation: A case report. *Thorac Cancer* (2021) 12:1770-4. doi: 10.1111/1759-7714.13941.

Urbán L, Dóczi R, Vodicska B, Tihanyi D, Horváth M, Kormos D, et al. Major clinical response to afatinib monotherapy in lung adenocarcinoma harboring EGFR exon 20 insertion mutation. *Clin Lung Cancer* (2021) 22:e112-e5. doi: 10.1016/j.cllc.2020.09.005.

Wang X, Huang L, Cai J, Liu A. A novel KIF5B-EGFR fusion variant in non-small-cell lung cancer and response to afatinib: a case report. *Onco Targets Ther* (2021) 14:3739-44. doi: 10.2147/ott.S313896.

Wei Y, Cui Y, Guo Y, Li L, Zeng L. A lung adenocarcinoma patient with a rare EGFR E709_T710delinsD mutation showed a good response to afatinib treatment: a case report and literature review. *Front Oncol* (2021) 11:700345. doi: 10.3389/fonc.2021.700345.

Wu SG, Yu CJ, Yang JC, Shih JY. The effectiveness of afatinib in patients with lung adenocarcinoma harboring complex epidermal growth factor receptor mutation. *Ther Adv Med Oncol* (2020) 12:1758835920946156. doi: 10.1177/1758835920946156.

Xu J. Non-small cell lung cancer (NSCLC) patients with rare epidermal growth factor receptor (EGFR) mutations in exons 18 and 19 benefit from treatment with EGFR tyrosine kinase inhibitors. Poster presented at the 2020 World Conference on Lung Cancer Singapore, January 28-31, 2021.

Yang G, Li J, Xu H, Yang Y, Yang L, Xu F, et al. EGFR exon 20 insertion mutations in Chinese advanced non-small cell lung cancer patients: Molecular heterogeneity and treatment outcome from nationwide real-world study. *Lung Cancer* (2020) 145:186-94. doi: 10.1016/j.lungcan.2020.03.014.

Yang X, Huang C, Chen R, Zhao J. Resolving resistance to osimertinib therapy with afatinib in an NSCLC patient with EGFR L718Q mutation. *Clin Lung Cancer* (2020) 21:e258-e60. doi: 10.1016/j.cllc.2019.12.002.

Zhang C, Lin L, Zuo R, Wang Y, Chen P. Response to tyrosine kinase inhibitors in lung adenocarcinoma with the rare epidermal growth factor receptor mutation S768I and G724S: A case report and literature review. *Thorac Cancer* (2020) 11:2743-8. doi: 10.1111/1759-7714.13606.

Zhang X, Jiang W, Yang N, Zhang Y. Afatinib response in a lung adenocarcinoma with novel compound S720F+L861R mutation in EGFR. *Lung Cancer* (2020) 148:170-2. doi: 10.1016/j.lungcan.2020.07.002.

Zhao H. Afatinib as a potential therapeutic option for non small cell lung cancer patients with EGFR G724S. Poster presented at the 2020 World Conference on Lung Cancer Singapore, January 28-31, 2021.

Zhao L, Wang Z, Du H, Chen S, Wang P. Lung adenocarcinoma patient harboring EGFR-KDD achieve durable response to afatinib: a case report and literature review. *Front Oncol* (2021) 11:605853. doi: 10.3389/fonc.2021.605853.

Zhao Y, Chen Y, Huang H, Li X, Shao L, Ding H. Significant benefits of afatinib and apatinib in a refractory advanced NSCLC patient resistant to osimertinib: a case report. *Onco Targets Ther* (2021) 14:3063-7. doi: 10.2147/ott.S300556.

Zhao Y, Zhai L, Deng L, Halmos B, Cheng H. Efficacy of osimertinib in afatinib-resistant lung cancer harboring uncommon EGFR mutations: case report and literature review. *Clin Lung Cancer* (2021) 22:e466-e9. doi: 10.1016/j.cllc.2020.06.017.

Zhu N, Dong C, Weng S, Yuan Y, Yuan Y. A patient of advanced NSCLC with a new EGFR exon 19 insertion mutation and its response to EGFR-TKIs. *J Coll Physicians Surg Pak* (2019) 29:S126-s8. doi: 10.29271/jcpsp.2019.12.S126.

Zöchbauer-Müller S, Kaserer B, Prosch H, Cseh A, Solca F, Bauer MJ, et al. Case report: afatinib treatment in a patient with NSCLC harboring a rare EGFR exon 20 mutation. *Front Oncol* (2020) 10:593852. doi: 10.3389/fonc.2020.593852.
